# Metabolism and memory: α-synuclein level in children with obesity and children with type 1 diabetes; relation to glucotoxicity, lipotoxicity and executive functions

**DOI:** 10.1038/s41366-022-01222-z

**Published:** 2022-09-24

**Authors:** Nouran Yousef Salah, Sara Ibrahim Taha, Safeya Hassan, Mai Seif ElDin Abdeen, Mostafa Ahmad Hashim, Rana Mahmoud

**Affiliations:** 1 Department of Pediatrics, Faculty of medicine, Ain shams University, Cairo, Egypt; 2 Department of Clinical Pathology, Faculty of medicine, Ain shams University, Cairo, Egypt; 3 Department of Psychiatry, Faculty of medicine, Ain shams University, Cairo, Egypt

**Keywords:** Diabetes, Obesity

## Abstract

**Background/Objectives:**

Children with obesity and those with type 1diabetes (T1D) exhibit subtle neurocognitive deficits, the mechanism of which remains unknown. α-synuclein plays a fundamental role in neurodegeneration. Moreover, its role in glucose and lipids metabolism is emerging. This study aims to assess whether α-synuclein is correlated with the degree of neurodegeneration in children with obesity and those with T1D in comparison to healthy controls and correlate it to various neurocognitive and metabolic parameters.

**Subjects/Methods:**

Forty children with obesity, 40 children with T1D and 40 matched-healthy controls were assessed for anthropometric measurements and blood-pressure. Cognitive evaluation was performed using Stanford–Binet scale and Barkley Deficits in Executive Functioning (EF) Scale-Children and Adolescents. α-synuclein, fasting lipids and glucose were measured with calculation of the homeostatic model of insulin-resistance and estimated-glucose disposal rate.

**Results:**

Children with obesity and those with T1D had significantly higher α-synuclein (*p* < 0.001) and total EF percentile (*p* = 0.001) than controls. α-synuclein was negatively correlated to total IQ (*p* < 0.001 and *p* = 0.001), and positively correlated with total EF percentile (*p* = 0.009 and *p* = 0.001) and EF symptom count percentile (*p* = 0.005 and *p* < 0.001) in children with T1D and obesity, respectively. Multivariate-regression revealed that α-synuclein was independently related to age (*p* = 0.028), diabetes-duration (*p* = 0.006), HbA1C% (*p* = 0.034), total IQ (*p* = 0.013) and EF symptom count percentile (*p* = 0.003) among children with T1D, and to diastolic blood-pressure percentile (*p* = 0.013), waist/hip ratio SDS (*p* = 0.007), total EF percentile (*P* = 0.033) and EF symptom count percentile (*p* < 0.001) in children with obesity.

**Conclusion:**

α-synuclein could have a mechanistic role in neurocognitive deficit among children with obesity and T1D.

## Introduction

Growing evidence suggests a link between obesity, diabetes and cognitive decline in adulthood. Associations were found between obesity, diabetes and risk of cerebral neurodegeneration including Alzheimer’s disease and Parkinsonism [1, 2]. Diabetes and obesity are now considered among the modifiable risk factors of dementias including Alzheimer’s disease [3].

Cognitive impairment was found to be prevalent among elderly with type 1 diabetes (T1D) and type 2 diabetes (T2D) [4, 5], with HbA1C correlating with poor cognitive functions and severity of dementia [6, 7]. In addition, studies suggest that obesity is associated with cognitive deficits, specifically in executive functioning (EF) skills [8]. Obesity has been shown to increase the risk of dementia, independent of diabetes [9]. Nevertheless, evidence has characterized obesity as a risk factor for the development of Alzheimer disease and Parkinsonism [10]. Some studies even proposed that weight reduction is associated with cognitive improvement [11].

Nonetheless, the relationship between obesity, diabetes and cognitive function in children is not as well established as in adults. Studies demonstrated subtle neurocognitive and pathological brain changes in children with TID [12]. Children with obesity and those with T1D are found to exhibit intelligence quotient (IQ) scores that are within normal range but significantly lower than healthy controls [13–15]. However, data about the cognitive and scholastic achievement in children with obesity are controversial [16, 17]. Although mild, neurocognitive deficits in children with obesity and those with T1D can impact the children learning and skills development and quality of life [18].

Emerging literature highlight the association between adipokines, gut hormones and cognitive functions [19]. α-synuclein; a 14 kDa protein highly expressed in the presynaptic terminals of the brain, regulating neurochemicals synthesis and synaptic vesicles exocytosis, namely the dopamine is one of such adipokines [20]. Intracellular deposition of α-synuclein has a fundamental role in several neurodegenerative diseases like Alzheimer’s disease and Lewy body disease [21].

However, it has been shown that the α-synuclein is expressed not only in neurons but also in various tissues including liver, spleen, blood, skeletal muscles and pancreatic β cells [22].

Moreover, α-synuclein was recently found to be involved in glucose homeostasis, where it enhances glucose uptake in the adipocytes, hepatocytes and myocytes, via activation of phosphatidylinositol 3kinase (PI3K) pathway, responsible for the metabolic action of insulin. Furthermore, it is involved in feedback control of insulin secretion through modulating the Kir 6.2 subunit of the ATP-sensitive potassium channel (K-ATP) in the pancreatic β cells [23]. Animal studies suggest that diet-induced obesity may be an environmental risk factor for the development of alpha-synucleinopathies [24].

Hence, the aim of this study was to assess serum α-synuclein level in children with obesity and those with T1D as a neurodegeneration biomarker in comparison to healthy controls and investigate its association with metabolic parameters and neurocognitive skills, specifically EF.

## Materials and methods

### Participants

Forty children with T1D were recruited from the regular attendees of the pediatric diabetes unit and 40 children with obesity were recruited from the pediatric obesity clinic, pediatric Hospital, Ain Shams University.

Forty apparently healthy Egyptian children were included as a control group from the siblings of attendees of the outpatients’ clinic. They were matched for age and gender. Participants were selected by simple random sampling.

Patients were categorized to T1D according to the criteria of International Society of Pediatric and Adolescent Diabetes (ISPAD) 2018 [25]. Diagnosis of obesity was defined as body mass index (BMI) > 95th percentile for age and gender [26].

Exclusion criteria included obese children with T1D, patients with comorbid neuropsychiatric illness (e.g., autism, epilepsy) and patients with other types of diabetes mellitus (e.g., type 2 diabetes mellitus).

Sample size: Using G* power program for sample size calculation, assuming that there would be 25% difference in the serum α-synuclein level between children with obesity, children with T1D, and healthy controls, a total sample size of 120 participants (40 per group) will be needed after taking in consideration 10% drop out rate.

### Ethical considerations

The study protocol was approved by the Ethical Committee of Ain Shams University with an approval number R 28/2021 and an informed consent was obtained from each patient or their legal guardians before participation.

### Procedure

#### Clinical assessment

All subjects involved in the study were subjected to the following:

1. A pre-formed detailed medical history sheet was used to record socio-demographic information and the participants’ clinical history.
  In children with T1D; special emphasis was done on the disease duration, regimen of insulin therapy (basal bolus or continuous subcutaneous insulin infusion), median self-monitoring of blood glucose (SMBG) per week and history of acute complications i.e. frequency of clinically significant hypoglycemia/ month and diabetic ketoacidosis (DKA) / year.
  Assessment of family socioeconomic level was done using the validated arabic socioeconomic level scale for health research in Egypt. It is a scale with 7 domains with a total score of 84 [26].
2. Physical examination was done, including.

- Anthropometric measures, including weight in kilograms (Kg) and height in centimeters (cm) and were plotted according to standard deviation scores (SDS) according to age and gender. BMI was assessed based on WHO recommendations [27].
  Waist circumference was measured midway between the lowest rib and the top of the iliac crest, while hip circumference was measured in a horizontal plane at the extension of the buttocks, and waist / hip ratio was calculated as well as the corresponding SDS scores [28].
  Sexual maturity assessment was done using Tanner staging [29].
- Blood pressure (BP) was measured manually, two consecutive times, by a sphygmomanometer in the right arm of a relaxed, seated child with comparison of values to normal reference percentiles [30].

#### Biochemical assessment

1. Serum α-synuclein level was assessed using a human ELISA kit supplied by BT Lab (Bioassay Technology Laboratory, Shanghai, China), with a detection range of 10 pg/ml− 1000 pg/ml and a sensitivity of 5.15 pg/ml.
2. Peripheral venous blood samples were collected in the morning from all patients after an overnight fast and separated on ethylene diamine tetraacetic acid (1.2 mg/mL) for analysis of HbA1c.
3. Fasting blood glucose was measured by SYNCHRON CX-9 autoanalyzer and fasting insulin was measured by an immunometric, chemiluminescent assay on IMMULITE Autoanalyzer (Siemens Medical Solution Diagnostics, Los Angeles, USA).
4. Homeostasis model assessment for insulin resistance (HOMA-IR) was calculated as follows: HOMA-IR = fasting glucose in millimoles per liter × fasting insulin in milli-international units per liter/22.5 A value of > 2.7 was used as cut off value for insulin resistance in children and adolescents [31].
5. For children with T1D the estimated glucose disposal rate (eGDR) was used to define insulin resistance using the following equation 21.158- (0.09 × waist circumference in cm) - (3.407 × hypertension) - (0.551 × HbA1C%) with a cut off value of 8.16 mg/kg/min [32, 33].
6. Fasting serum triglycerides (TG) and total cholesterol (TC) were assessed by quantitative enzymatic colorimetric technique (Bio Merieux-Diagnostic Chemicals Ltd., Charlottetown,CA,USA). Serum high-density lipoprotein (HDL) was measured by the phosphotungstate precipitation method (Bio Merieux kit, Marcyl’Etoile, Craponne, France). LDL cholesterol was calculated by Friedewald’s formula [34].

#### Psychometric assessment

1. The Stanford–Binet intelligence test:
  Psychometric evaluation of both patients and controls by Stanford-Binet Scale 4th edition was done to assess general intelligence quotient [35]. Stanford-Binet Scale measures intelligence and cognitive abilities in children and adults; from age 2 through 23 years; providing a full-scale IQ. It is composed of five components: fluid reasoning, knowledge, quantitative reasoning, visual-spatial processing, and working memory (subtests are grouped together to form one of the two domains or one of the five factor indices: The two domains or the five factor indexes are added together to obtain the full-scale IQ score. The Cronbach’s alpha coefficient for the full-scale IQ scores is 0.97 to 0.98 and for the verbal and nonverbal IQ scores is 0.95–0.96. Arabic version was used [36].
2. Barkley Deficits in Executive Functioning Scale--Children and Adolescents (BDEFS-CA)- Parent-report rating scale:

The long form of the BDEFS-CA long form provides a valid assessment of EF *deficits* in daily life activities with an age range of 6–17 years. The scale demonstrates satisfactory reliability and validity [37]. Arabic version was used [38].

The long form of BDEFS-CA is scored by calculating the totals for each of the five scales: self-management to time, self-organization and problem-solving, self-restraint, self- motivation, and self-regulation of emotion. Additionally, the instrument yields a total executive functioning summary score (the total of the five scales) and symptom count (number of items rated as occurring often or very often). Results from the BDEFS-CA can be interpreted using four different approaches by: 1) interpreting the meaning of each scale separately by identifying high subscale scores; 2) making normative comparisons (percentile scores based on sex and age group; 3) conducting risk analysis to aid in clinical interpretation with respect to major domains of life activity beyond the BDEFS subscales; and 4) assessing change in patients resulting from treatment. Norms (percentiles) in the BDEFS-CA are provided for boys and girls in two age ranges: 6–11 years and 12–17 years. The higher the score the more EF *deficits* present. According to the percentiles score, children with score ≤ 92: no affection, those with score 93–95: mild affection, those scoring 96–98: moderate affection, and children scoring ≥ 99: severe affection [37].

### Statistical analysis

Data were collected, coded and entered to the Statistical Package for Social Science (IBM SPSS), version 23.0 (Armonk, New York: IBM Corporation, released 2015). The quantitative data were presented as mean and standard deviations when parametric and median, inter-quartile range (IQR) when non-parametric. Qualitative variables were presented as number and percentage. The comparison between groups regarding qualitative data was done using Chi-square test. The comparison between more than two groups regarding quantitative data and parametric distribution was done by using One Way ANOVA test followed by post hoc analysis using LSD test while the comparison between more than two groups with non-parametric distribution was done by using Kruskall-Wallis test followed by post hoc analysis using Mann-Whitney test. Spearman correlation coefficients were used to assess the correlation between two quantitative parameters in the same group.

Multivariate linear regression analysis was used to assess predictors of α-synuclein level in each group. The confidence interval was set to 95% and the margin of error accepted was set to 5%. So, the p-value was considered significant at the level of < 0.05.

## Results

One hundred-twenty randomly selected pediatric patients and healthy controls who met the eligibility criteria were included in the study. There were no dropouts. Participants were divided into 3 equal groups; 40 children with obesity, 40 non-obese children with T1D and 40 age and gender matched healthy children who served as control group.

### Clinical characteristics of the obesity and the T1D groups

The mean age of the studied children with obesity was 12.7 ± 3.1 years, while that of the T1D group was 13.6 ± 2.5 years and the control group was 13.3 ± 2.2 years. Females comprised 64.2% of the entire studied cohort.

No significant difference was found between the three study groups as regards Tanner staging and the socioeconomic scale.

Regarding children with obesity, their median BMI SDS (IQR) was 3.25 (2.76–4.31), their mean waist/ hip ratio was 0.9 ± 0.1 and their mean HOMA-IR was 3.2 ± 1.6.

As for children with T1D, thirty seven children (92.5%) were on basal bolus insulin regimen and three children (7.5%) were on continuous subcutaneous insulin infusion. Their mean HbA1C was 9.2 ± 2.2 %, their mean fasting blood glucose was 119.5 ± 30.1 mg/dl and their median SMBG (IQR) reading over 1 week was 151.55 ± 33.14 mg, range 95–216. The frequency of clinically significant hypoglycemia /month among these children ranged from 0–3 and the median frequency of DKA / year ranged from 0–4. Regarding insulin sensitivity, their mean e-GDR was 9.4 ± 1.6.

Upon comparing the three study groups, systolic and diastolic blood pressure percentiles, fasting cholesterol, triglycerides and LDL were highest in the obese group followed by the T1D group than the controls (*p* < 0.05), [Table 1].

**Table 1:** Demographic and clinical data of the studied children with obesity, children with T1D, and controls.

	Obese group *n* = 40	T1D group *n* = 40	Control group *n* = 40	Test value	*P*-value	Post hoc analysis		
						P1	P2	P3
Age (years) Mean±SD	12.7 ± 3.1	13.6 ± 2.5	13.3 ± 2.2	1.364	0.259^ο^	-	-	-
Gender								
Females	26 (65%)	25 (62.5%)	26 (65%)	0.072	0.964^c^	-	-	-
Males	14 (35%)	15 (37.5%)	14 (35%)					
Socioeconomic level scale Mean ± SD	53.80 ± 8.62	55.20 ± 7.07	56.55 ± 4.71	1.549	0.217 ^ο^			
Weight SDS Median (IQR)	2.9 (2.1–3.7)	0.6 (−0.3 to 1.2)	−0.7 (−1.2 to −0.4)	118.948	**<** **0.001ǂ**	**<** **0.001**^×^	**<** **0.001**^×^	**<** **0.001**^×^
Height SDS Median (IQR)	0.4 (−0.8–0.9)	−0.7 (−1.5 to 0.1)	−0.8 (−1.1 to 0.2)	3.801	**0.025ǂ**	0.312^×^	**0.016**^×^	**0.013**^×^
BMI SDS Median (IQR)	3.25 (2.76–4.31)	1.0 (0.3–1.42)	−0.11 (−0.74 to 0.14)	264.749	**<** **0.001ǂ**	**<** **0.001**^×^	**<** **0.001**^×^	**<** **0.001**^×^
Waist circumference (cm) Mean ± SD	87.6 ± 13.8	73.9 ± 11.4	57.5 ± 6.0	76.687	**<** **0.001** ^ο^	**<** **0.001**^×^	**<** **0.001**^×^	**<** **0.001**^×^
Waist / Hip ratio Mean ± SD	0.9 ± 0.1	0.8 ± 0.1	0.9 ± 0.0	35.960	**<** **0.001** ^ο^	**<** **0.001**^×^	**<** **0.001**^×^	**<** **0.001**^×^
Tanner Stage 1	11 (27.5%)	12 (30.0%)	11 (27.5%)					
2	10 (25.0%)	11 (27.5%)	12 (30.0%)					
3	6 (15.0%)	6 (15.0%)	5 (12.5%)	1.333	0.995 ^c^			
4	7 (17.5%)	4 (10.0%)	5 (12.5%)					
5	6 (15.0%)	7 (17.5%)	7 (17.5%)					
Systolic blood pressure percentile Mean ± SD	84.3 ± 18.9	67.4 ± 15.4	60.0 ± 17.5	20.654	**<** **0.001** ^ο^	**0.049**^×^	**<** **0.001**^×^	**<** **0.001**^×^
Diastolic blood pressure percentile Mean ± SD	90.8 ± 10.2	66.3 ± 14.9	60.0 ± 17.5	49.982	**<** **0.001** ^ο^	0.090^×^	**<** **0.001**^×^	**<** **0.001**^×^
HbA1C (%) Mean ± SD	5.6 ± 0.6	9.2 ± 2.2	4.5 ± 0.5	132.494	**<** **0.001** ^ο^	**<** **0.001**^×^	**<** **0.001**^×^	**<** **0.001**^×^
Fasting glucose (mg/dl) Mean ± SD	86.6 ± 8.2	119.5 ± 30.1	81.3 ± 5.7	51.269	**<** **0.001** ^ο^	**<** **0.001**^×^	**0.001**^×^	**<** **0.001**^×^
HOMA-IR Mean ± SD	3.2 ± 1.6	9.2 ± 4.6	0.7 ± 0.3	94.667	**<** **0.001** ^ο^	**<** **0.001**^×^	**<** **0.001**^×^	**<** **0.001**^×^
Cholesterol (mg/dl) Mean ± SD	172.5 ± 31.2	149.8 ± 36.5	149.8 ± 18.9	7.779	**0.001** ^ο^	0.998^×^	**<** **0.001**^×^	**0.004**^×^
HDL (mg/dl) Mean ± SD	45.1 ± 15.1	37.5 ± 5.7	49.9 ± 21.4	6.534	**0.002** ^ο^	**0.001**^×^	**0.004**^×^	0.248^×^
LDL (mg/dl) Mean ± SD	110.3 ± 25.9	78.9 ± 32.2	90.4 ± 19.5	14.813	**<** **0.001** ^ο^	**0.001**^×^	0.056^×^	**<** **0.001**^×^
Triglycerides (mg/dl) Mean ± SD	102.5 ± 43.6	88.9 ± 31.9	92.5 ± 10.4	1.964	0.145 ^ο^	0.496^×^	0.163^×^	0.116^×^
α-synuclein (pg/ml) Median (IQR)	600 (400–1080)	120 (100–160)	30 (30–40)	106.468	**<** **0.001** ^ο^	**<** **0.001**^×^	**<** **0.001**^×^	**<** **0.001**^×^

*SDS* Standard deviation score, *BMI* Body mass index, *HbA1C* Glycated hemoglobin, *HOMA-IR* Homeostatic model of insulin resistance, *HDL* High density lipoproteins, *LDL* Low density lipoproteins.

^c^Chi-square test, ^ο^One Way ANOVA, ^**ǂ**^Kruskall-Wallis, ^×^LSD test, ^#^Mann-Whitney test, *P* < 0.05: significant.

P1: Comparison between children with T1D and controls.

P2: Comparison between children with obesity and controls.

P3: Comparison between children with obesity and those with T1D.

### Neurocognitive assessment and α-synuclein in children with obesity and children with T1D

**Fig. 1:**
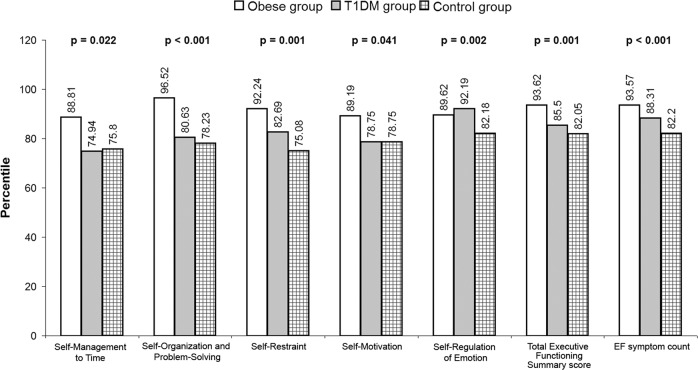
Comparison of executive functions (by BDEFS-CA) between children with obesity, children with T1D and healthy controls showing significantly increased total executive functioning summary score percentile and its subscales percentiles in children with obesity than those with T1D and controls.

Regarding the EF, the total executive functioning summary score percentile and it’s subscales were significantly impaired in the obesity and T1D groups than controls being highest in the obesity group, followed by the T1D group then the controls (*p* < 0.05), [Table 2]; except for the self-regulation of emotions which was highest in the T1D group followed by the obesity group then the controls (*p* = 0.002), [Fig. 1]. The increased executive functioning summary score percentile in children with obesity than those with T1D and controls coincides with similar elevation in the α-synucelin level, which was found to be highest in the obesity group followed by the T1D group then the controls (*p* < 0.001), [Table 1].

**Table 2:** Comparing cognitive functions between children with obesity, children with T1D and healthy controls.

	Obese group *n* = 40	T1D group *n* = 40	Control group *n* = 40	*P*-value^O^
Stanford–Binet Intelligence Scale Total IQ Mean ± SD	84.7 ± 10.9	84.5 ± 7.1	88.7 ± 7.3	0.107
BDEFS-CA				
Self-Management to Time percentile Mean ± SD	88.8 ± 4.6	74.9 ± 28.7	75.8 ± 17.5	**0.022**
Self-Organization and Problem-Solving percentile Mean ± SD	96.5 ± 1.4	80.6 ± 21.3	78.2 ± 15.9	**<** **0.001**
Self-Restraint percentile Mean ± SD	92.2 ± 4.5	82.7 ± 21.7	75.1 ± 18.7	**0.001**
Self-Motivation percentile Mean ± SD	89.2 ± 4.4	78.8 ± 20.3	78.8 ± 17.5	**0.041**
Self-Regulation of Emotion percentile Mean ± SD	89.6 ± 4.5	92.2 ± 8.2	82.2 ± 12.6	**0.002**
Total executive functioning summary score percentile Mean ± SD	93.6 ± 2.6	85.5 ± 15.2	82.1 ± 11.1	**0.001**
EF symptom count percentile Mean ± SD	93.6 ± 2.4	88.3 ± 10.8	82.2 ± 12.0	**<** **0.001**

*BDEFS-CA* Barkley Deficits In Executive Functioning Scale—Children and Adolescents.

^ο^One Way ANOVA, *P* < 0.05: significant.

Bold values indicates statistical significant *P* values.

As for cognition, children with T1D had significantly lower total IQ than controls (*p* = 0.011). However, no statistically significant difference was found between children with obesity and controls (*p* = 0.255).

### α-synuclein and neurocognitive data

On correlating α-synuclein with all domains of neurocognitive functions in patients’ groups, we found a significant negative correlation between α-synuclein and total IQ (*P* = 0.001 and *P* < 0.001) and a significant positive correlation with total executive functions percentile (*P* = 0.001 and *P* = 0.009) and executive functions symptom count percentile (*P* < 0.001 and *P* = 0.005) among the studied children with obesity and T1D, respectively [Table 3].

**Table 3:** Correlation of factors associated with α-synuclein in the children with T1D and children with obesity.

	α-synuclein level			
	Children with obesity *n* = 40 (33.3%)		Children with T1D *n* = 40 (33.3%)	
	*r*	*p*-value	*r*	*p*-value
Age (Years)	0.091	0.578	0.357	**0.024**
Clinical data				
Socioeconomic level scale	−0.258	0.108	−0.045	0.785
Duration of Disease (Years)	0.044	0.789	0.538	**<** **0.001**
Systolic blood pressure percentile	0.288	0.071	0.166	0.307
Diastolic blood pressure percentile	0.453	**0.003**	0.059	0.716
Weight SDS	0.135	0.406	0.239	0.137
Height SDS	−0.171	0.292	0.170	0.295
BMI	0.206	0.203	0.097	0.553
BMI SDS	0.186	0.251	0.285	0.074
Waist Circumference (cm)	−0.048	0.769	0.222	0.144
Waist/ hip ratio	0.427	**0.006**	0.187	0.248
Waist / hip Ratio SDS	0.535	**<** **0.001**	0.084	0.608
Tanner Stage	0.108	0.508	0.309	0.052
HbA1C (%)	0.037	0.823	0.498	**0.001**
Fasting glucose (mg/dl)	0.048	0.768	−0.247	0.124
Mean SMBG (mg/dl)			−0.076	0.643
Frequency of clinically significant hypoglycemia /month			−0.007	0.966
Frequency of DKA /year			−0.160	0.325
eGDR			−0.125	0.442
HOMA-IR	−0.086	0.596	−0.115	0.479
Cholesterol (mg/dl)	0.076	0.642	0.071	0.662
HDL (mg/dl)	−0.230	0.154	−0.029	0.860
LDL (mg/dl)	0.180	0.266	0.129	0.427
Triglycerides (mg/dl)	0.094	0.565	0.051	0.756
Cognitive data				
Total IQ	−0.530	**0.001**	−0.593	**<** **0.001**
BDEFS-CA				
Self-Management to Time percentile	0.147	0.366	0.279	0.295
Self-Organization and Problem-Solving percentile	0.521	**0.001**	0.174	0.519
Self-Restraint percentile	0.021	0.898	0.277	0.299
Self-Motivation percentile	0.204	0.206	0.376	0.151
Self-Regulation of Emotion percentile	0.048	0.766	0.476	0.062
Total executive functioning score percentile	0.523	**0.001**	0.525	**0.009**
EF symptoms count percentile	0.771	**<** **0.001**	0.662	**0.005**

*T1D* Type 1 diabetes, *SDS* Standard deviation score, *BMI* Body mass index, *HbA1C* Glycated hemoglobin, *eGDR* Estimated glucose disposal rate, *HOMA-IR* Homeostatic model of insulin resistance, *LDL* Low density lipoproteins, *HDL* High density lipoproteins, *IQ* Intelligence quotient, *EF* Executive functions.

Spearman correlation coefficients, *P* < 0.05: significant.

Bold values indicates statistical significant *P* values.

### α-synuclein and metabolic data (insulin resistance, lipid profile, and glycemic control)

Correlating α-synuclein with metabolic data showed that in children with obesity α-synuclein positively correlated with diastolic blood pressure percentile (*p* = 0.003), Waist/ hip ratio (*p* = 0.006) and Waist / hip ratio SDS (*p* = < 0.001). As for children with T1D, α-synuclein positively correlated with duration of disease (*p* = < 0.001) and HbA1C (*p* = 0.001).

Nonetheless, it was not correlated to HOMA-IR among children with obesity (*P* = 0.596) and children with T1D (*P* = 0.479), nor with e-GDR (*P* = 0.442) among children with T1D [Table 3].

The variables with a significant association with the dependent variable α-synuclein level were included in the multivariate regression analysis [Table 4]. In children with T1D, α-synuclein was found to be independently associated with age ((*p* = 0.028), duration of diabetes (*p* = 0.006), HbA1C% (*p* = 0.034), EF symptom count percentile (*p* = 0.003) and total IQ (*p* = 0.013). While in children with clinical obesity, it was independently associated with diastolic blood pressure (*p* = 0.013), waist to hip ratio SDS (*p* = 0.007), total EF score percentile (*p* = 0.033) and EF symptom count percentile (*p* < 0.001).

**Table 4:** Multivariate backward regression analysis for factors associated with α-synuclein among children with T1D and children with obesity.

	Unstandardized Coefficients		Standardized Coefficients	*t*	Significance
	B	SE	Beta		
Children with T1D *n* = 40 (33.3%)					
(Constant)	462.232	67.333		6.865	0.000
Age (years)	4.783	1.897	0.470	2.522	**0.028**
Duration of diabetes (years)	5.004	1.458	0.620	3.433	**0.006**
Total IQ	−2.082	0.706	−0.409	−2.950	**0.013**
EF symptom count percentile	1.422	0.368	0.553	3.860	**0.003**
HbA1C%	5.752	2.180	0.379	2.638	**0.034**
Children with obesity *n* = 40 (33.3%)					
(Constant)	1835.866	519.022		3.537	**0.001**
Diastolic blood pressure/ mmHg	8.548	3.236	0.233	2.642	**0.013**
Waist hip ratio SDS	89.950	30.900	0.266	2.911	**0.007**
Total EF summary score percentile	10.198	4.544	0.248	2.244	**0.033**
EF symptom count percentile	15.330	3.676	0.481	4.171	**<** **0.001**

*SDS* Standard deviation score, *EF* Executive functions, *T1D* Type 1 diabetes mellitus, *IQ* Intelligence quotient, *P* < 0.05: significant.

Bold values indicates statistical significant *P* values.

## Discussion

Diabetes and obesity have been suggested as risk factors for cerebral neurodegeneration in adults [39, 40]. Protein aggregation and neuro-inflammation are considered common dysregulated pathways in diabetes, obesity and neurodegenerative diseases [24]. However, there is a paucity of research on the interactive relationship between metabolism and cognition in the pediatric population. This study was set out to assess the role of α-synuclein in the interplay between metabolic derangement and neurodegeneration in children with T1D and those with obesity.

Children with obesity and those with T1D were found to have significant EF impairment as indicated by significantly higher total EF score with all its subscales compared to healthy controls. This EF impairment was more significant in those with obesity than those with T1D.

The vast majority of evidence about the association between DM and cognitive impairment come from T2D studies with only few studies focusing on the effect of hyperglycemia caused by T1D on cerebral neurodegeneration [41, 42]. Cognitive impairments and structural brain alterations were documented in adults with T1D [43]. Moreover, mild disruptions in cognitive function were found in children with T1D [44, 45]. A recent meta-analysis revealed that EFs are particularly affected in children with T1D [46]. Moreover, McNally and colleagues showed that EFs are related to glycemic control in people with T1D [47].

The mechanism of cognitive impairment among people with T1D remains to be unraveled. One of the proposed mechanisms explaining cognitive impairment in T1D on the cellular level is neuro-inflammation. Persistent hyperglycemia in T1D saturate mitochondrial respiration in endothelial cells, astrocytes and pericytes promoting reactive oxygen species (ROS) production and oxidative stress [48]. These ROS together with inflammatory cytokines interrupt the blood brain barrier integrity. Upon penetration by proinflammatory factors, microglia are activated, and neuroinflammation occur [49]. Neuroinflammation is a well-recognized feature of cerebral neurodegeneration.

The association between obesity and cognitive function in children/adolescents however remains unraveled.

In agreement with the current study, some studies show deficits in EF including impulsivity and poor inhibitory control when comparing children with obesity to non-obese children [50, 51]. A meta-analysis observed small-to-moderate negative associations between obesity and executive and reward-related performance, but not impulsivity in children [52]. One of the theories explaining the link between obesity and cognitive dysfunction is through lipotoxicity. Obesity is associated with chronically elevated levels of circulating free fatty acids which causes low-grade inflammation and plays an important role in insulin resistance. In addition, positron emission tomography studies have shown fatty acid uptake by the brain in people with metabolic syndrome. The accumulation of saturated fatty acids in the brain causes activation of the immune system through the toll-like receptor 4 (TLR4) proteins that detects lipopolysaccharides. TLR4 activation leads to the generation of cytokines in astrocytes [22]. Moreover, excessive nutrition can cause abnormalities in the hypothalamus through insensitivity to insulin and leptin hormones, as well as atrophy in the hippocampus [53]. Energy imbalance, altered circuits and signaling pathways, generalized inflammatory state, oxidative stress, impaired cerebrovascular blood flow, insulin resistance and lipid associated neurotoxicity are among the suggested theories [54].

The relation of obesity/ diabetes and cognitive impairment is thought to be bidirectional. In one hand, the level of EF affects treatment adherence and self-management in T1D/obesity. On the other hand problematic adherence, may lead to poor glycemic control/ dysregulated eating behavior, which, in turn, may negatively impact EF [50, 52]. New biomarkers are needed to unravel the patho-mechanism of cognitive impairment among children with T1D and those with obesity and to allow early detection and follow up of cognitive impairment among those children.

α-synuclein is a well-known biomarker of neurodegeneration that deposits in cerebral neuronal cell bodies of people with Lewy body dementia’s including parkinsonism [55]. In addition, α-synuclein is expressed in peripheral tissues including pancreatic islets and skeletal muscle [56]. However, the role of α-synuclein in the interplay between metabolic derangement and cerebral neuro-degeneration is to be assessed.

In the current study, α-synuclein was highest in children with obesity followed by children with T1D then controls. This elevated level of α-synuclein in children with obesity coincides with the EF deficit in this cohort. Studies on α- synuclein relation to EF in obesity and T1D remain scarce. Animal studies stated that high fat diet induces obesity and glucose intolerance in a transgenic mouse model for α-synucleinopathy, and thereby leads to earlier α -synucleinopathy and astrogliosis. They found that transgenic mice fed on high fat diet develop Lewy body-like aggregations in neuronal cell bodies at the age of 16 months compared to 20 months in those fed on standard diet [24]. Another murine study showed increased α-synuclein mRNA expression in the midbrain of obese mice. They assumed that elevated α-synuclein might be the cause of neurodegeneration associated with obesity [57].

Pathological α-synuclein deposits were found in the pancreatic beta cells of 93% of people with Parkinsonism and 68% of people with T2D compared to 17% of controls [58]. Similarly, increased deposition and phosphorylation of α-synuclein was observed in the pancreatic islets of murine models of T2D [59].

### α-synuclein and neurocognitive data

In the current study, α-synuclein was found to be negatively correlated to total IQ and positively correlated with total EF deficits among children with obesity / T1D. In the obese group α-synuclein also positively correlated with self-organization and problem-solving subscales, yet it was not of significance in multi-regression analysis.

Adult studies suggest a casual role of α-synuclein in Parkinsonism and cognitive deficit [60]. Studies in pediatric population however are scarce. Plasma α-synuclein was found to be elevated in children with autistic spectrum disorder and correlating with disease severity and intellectual impairment [61]. Moreover, serum α-synuclein was significantly increased in children with epilepsy and with acquired demyelinating disorders of the CNS [62]. These two studies may imply that serum α-synuclein is a potential biomarker of neurodegenerative processes. However, to the best of our knowledge no previous studies addressed the association of α-synuclein with neurodegeneration in pediatric population diagnosed with obesity/ T1D.

### α-synuclein and metabolic data

Children with T1D showed a significant positive relationship between the α-synuclein serum level and age, duration of diabetes and HbA1C.

In the same line, the NEDICES study found that diabetes duration was an important factor in the association between parkinsonism and diabetes [63]. The deleterious effect of chronic hyperglycemia on neurons in diabetes is well known. Glucose uptake by neurons is independent of insulin. Thus, neurons are particularly exposed to fluctuating glucose levels. Intracellular glucose is normally phosphorylated to glucose-6-phosphate to enter glycolysis or the pentose phosphate pathway. However, hyperglycemia leads to diverging metabolic routes giving rise to reactive dicarbonyl species and advanced glycation end products (AGE) [62].

While hyperglycemia is reversible, glycation of tissue proteins accumulates over lifetime, contributing to diabetes related chronic complications. α-synuclein is a long-lived protein and, therefore, is likely to be glycated over time. Glycated α-synuclein is more prone to oligomerization and aggregation [64].

It worth noting, that α-synuclein was not correlated to HOMA-IR among children with obesity / T1D, nor with e-GDR among children with T1D. Moreover, comparison of children with T1D and those with obesity with and without insulin resistance revealed no significant association between insulin resistance and α-synuclein, total IQ and executive functions score percentile. In contrast to the current study, Rodriguez-Araujo et al. found an inverse correlation of α-synuclein levels with insulin resistance indicators (body mass index, HOMA-IR) and a weaker correlation with DBP and age [65]. However, this study was on a sample of healthy adults with no insulin resistance, diabetes or obesity.

In children with obesity, although α-synuclein was not correlated to HOMA-IR, it was positively correlated to diastolic blood pressure percentile and waist hip ratio SDS. Similar studies were hard to find to compare with. However, in support the current results, a longitudinal study of 6582 participants showed that individuals with high waist circumference have a three-fold risk of developing dementia compared with controls [9]. In addition, large waist-hip ratio was found to be associated with decreased hippocampal volume [10]. In line with these results, a murine study showed considerable individual inter-animal variability in the total brain stem lysate phosphor-Akt levels. However, they found significant reduction in in total brain stem lysate phospho-Akt levels in 17 month-old transgenic mice fed on high fat diet compared to standard diet which could point to insulin resistance as a contributing factor for neurodegeneration in transgenic obese mouse [24].

The link between diabetes and obesity, α-synuclein and neurodegenration remains obscure. A neural gut-brain axis theory was postulated suggesting a centripetal spread of α-synuclein pathology from the enteric nervous system (ENS) to the brain. Pathological α-synuclein is assumed to act as proteinaceous nuclei which recruit endogenous cellular or post-translationally modified α-synuclein and integrate it into their own misfolded polymeric aggregate structure leading to α-synuclein deposition [66]. This α-synuclein deposition plays a fundamental role in central and peripheral neurodegeneration. Additionally, α-synuclein is found in various tissues including the liver, kidney, spleen, red blood cells, cardiomyocytes and pancreatic β cells [67]. Studies reported concomitant accumulation of α-synuclein in the insulin secretoy granules in the pancreatic islets and brain regions in monkeys with T2DM [59]. In one hand, α-synuclein is thought to inhibit insulin secretion by binding to KATP channels, resulting in insulin reduction, aggravating the process of diabetes [68]. On the other hand, impaired insulin signaling has been shown to influence the lysosomal system contributing to α-synuclein aggregation in the pancreatic islets and also in the brain [59].

### Limitations

Several important limitations should be borne in mind when interpreting these results: First, the cross-sectional design of the study may undermine its ability to affirm causal inferences. Second, the relatively small number of patients enrolled may limit generalizability of results. Third, detection of α-synuclein in the body fluid differs from α-synuclein in solid tissue samples of the enteric and autonomic nervous system, but it offers some potential as a surrogate marker of brain synucleinopathy.

A prospective approach will give a better understanding of the causality relationship between serum α-synuclein, various metabolic parameters and neurodegeneration among children with T1D/obesity.

### Implications

Knowing that most beneficial neuroprotective effects might only be achieved in early stages of any degenerative processes, identifying of risky children is of utmost importance. Therefore, implementation of neuroprotective measures to stop the neurodegenerative processes in exposed children is mandatory.

Additional therapeutic approaches may be needed to aid these children not only to reduce their obesity and glycemic derangements, but also to enhance their EF skills. Moreover, children with clinically significant EF deficits may benefit from additionally provided family support. By sharing responsibility for treatment, parents may be able to compensate for impairment in EF.

## Conclusion

In conclusion, serum α-synuclein is increased among children with obesity and those with T1D than controls; being highest in the obese group. This increase is correlated with executive functions impairment among these cohorts. Diastolic blood pressure and waist hip ratio are the most significant independent metabolic parameters associated with α-synuclein increase among children with obesity; while diabetes duration, age and HbA1C are the most independent parameters among children with T1D. Thus, serum level of α-synuclein is a potential biomarker for cognitive affection and neurodegeneration in children with obesity and those T1D. As such, α-synuclein may provide new avenues to novel therapeutics in neurodegeneration, obesity and diabetes. Studies should address the role of various anti-diabetic drugs including metformin, anti-obesity drugs and anti-glycation drugs as potential therapy for cerebral neurodegeneration and their impact on the prevention and treatment of cerebral neurodegeneration.

## Data Availability

Data will be available upon request from the corresponding author.
